# Reevolution of Tissue Regeneration: From Recent Advances in Adipose Stem Cells to Novel Therapeutic Approaches

**DOI:** 10.1155/2021/2179429

**Published:** 2021-02-11

**Authors:** Francesco De Francesco, Csaba Matta, Michele Riccio, Andrea Sbarbati, Ali Mobasheri

**Affiliations:** ^1^Department of Plastic Reconstructive Surgery and Hand Surgery, Azienda Ospedaliero Universitaria Ospedali Riuniti, 60126 Ancona, Italy; ^2^Department of Anatomy, Histology and Embryology, Faculty of Medicine, University of Debrecen, Debrecen, Hajdu-Bihar, Hungary; ^3^Department of Neurosciences, Biomedicine and Movement Sciences, Anatomy and Histology Division, University of Verona, 37134 Verona, Italy; ^4^Research Unit of Medical Imaging, Physics and Technology, Faculty of Medicine, University of Oulu, PO Box 5000, FI-90014 Oulu, Finland; ^5^Department of Regenerative Medicine, State Research Institute Centre for Innovative Medicine, LT-08406 Vilnius, Lithuania; ^6^University Medical Center Utrecht, Department of Orthopedics, Rheumatology and Clinical Immunology, 508 GA, Utrecht, Netherlands

Basic, translational, and clinical research in the area of stem cell biology is currently aimed at investigating the ability of adipose stem cells (ASCs) to enhance tissue and organ regeneration and suggest that ASCs in 3D scaffolds may have potential for application in wound healing, orthopaedic tissue repair, and tissue reconstruction after surgery [1, 2]. The development of regenerative medicine strategies requires an appropriate phenotypically stable and well-characterized cell source and a 3-dimensional scaffold or “smart” biomaterial (novel “intelligent” biomaterial with appropriate physical properties that can support *in vivo* the commitment of ASCs), together with a suitable microenvironment to provide the necessary cues and signals for cell growth and tissue formation. Tissue reconstruction represents one of the most significant challenges in all surgical procedures. This special issue includes a collection of papers that have focused on these aspects in regenerative medicine and tissue engineering.

G. Storti and colleagues [3] focused their review on recent discoveries that have involved the use of ASCs in bone tissue engineering and analysed the interaction of ASCs with scaffolds that can support osteogenic commitment. Moreover, S.-J. Oh and colleagues [4] explored the therapeutic efficacy of ASCs and their secretome in auricular cartilage regeneration and highlighted that the expression of collagen type II, TGF, beta1, and IGF-1 was significantly higher after ASC injection in an auricular defect. M. Milewska and colleagues [5] demonstrated that copper sulphate supplementation of ASCs can have a beneficial effect on tendon regeneration but not inducing tenogenic differentiation and improve the recruitment of MSC to the site of injury and prevent the effects of inflammation oxidative stress. M. Neubauer and colleagues [6] demonstrated that blood products such as platelet-rich plasma (PRP), hyperacute serum, and standard fetal calf serum have a significant influence on the viability and differentiation potential of ASCs especially in an osteogenic and chondrogenic context. In addition, M. Conese and colleagues [7] showed that ASCs represented an alternative strategy to increase the healing rate of hard-to-heal wounds with the use of dermal matrix, collagen scaffolds, and platelet-rich plasma. Moreover, X. Qin and colleagues [8] demonstrated that ASCs rapidly promote fistula healing, and C. Hu and colleagues [9] showed that ASCs have a strong protective effect on degenerative diseases of the retina.

Tissue engineering represents a collection of technologies combining biomaterials and stem cells, provides tools for regenerative medicine, and is expanding tremendously from biomaterial science towards a genuine multidisciplinary area, integrating biology, medicine, and various engineering sciences. S. Zhou [10] prepared an acellular matrix using ASC sheets. This novel biomaterial possesses good recellularization capacity and excellent biocompatibility. Moreover, O.A. Mohiuddin and coworkers [11] showed that decellularized adipose tissue-derived hydrogel is a cytocompatible scaffold, which supports the adipogenic and osteogenic differentiation of ASCs, and J.J. dos Santos Machado et al. [12] performed an animal study using a solution containing 1% hyaluronic acid and ASC injected subcutaneously. The solution tested in this study did not result in systemic, biochemical, or anatomic alterations that could represent toxicity symptoms. Commonly utilized in vitro models employ human or mouse preadipocyte cell lines in a 2-dimensional (2D) format. However, 3D tissue engineering scaffolds are better able to mimic the in vivo cellular microenvironment and more effectively recapitulate the physiological setting, resulting in improved localization, attachment, proliferation, and differentiation of ASCs. R. Bender et al. [13] conducted a study using 3-dimensional cultures and ObaGel, a thermoresponsive gel with a structure that is driven by cell-mediated extracellular matrix remodelling, and demonstrated that a combination of primary SVF cells and ObaGel scaffolds can be used to create a 3D *in vitro* construct with functional properties mimicking a humanized adipose depot.

We know from these studies and from many other published studies that ASCs have regenerative potential and are used in a significant number of clinical trials with a good immunomodulatory effect. Despite these advances, the functional immunomodulatory capacity of these cells is not yet fully understood. H.Y. Zhang and colleagues [14] founded that ASCs with miR-129-5p knockdown exhibited enhanced immunosuppressive capacity, as evidenced by reduced expression of proinflammatory factors, with concurrent increased expression of inducible nitric oxide synthases (iNOS) and nitric oxide (NO) production. ASCs with miR-129-5p knockdown alleviated inflammatory bowel disease and promoted tumour growth *in vivo*. Moreover, J.S. Heo [15] examined the effect of MSC-induced macrophages on inflammation and immune modulation and proposed that MSC-induced macrophages may be used as a novel stem cell-based cell-free therapy for the treatment of immune-mediated inflammatory disorders.

The current special issue focused on the potential role of ASCs in tissue engineering and regenerative medicine, presenting new information about implantable biocompatible materials, and advancing the knowledge of ASCs and biomaterials with potential for further development along with future stem cell-based therapeutics and technology platforms.

*Francesco De Francesco* *Csaba Matta* *Michele Riccio* *Andrea Sbarbati* *Ali Mobasheri*
